# Supplementary figures and images for: EGR1 regulates oral epithelial cell responses to Candida albicans via the EGFR- ERK1/2 pathway
Source: Virulence. 2024 Dec 5;15(1):2435374. doi: 10.1080/21505594.2024.2435374 (PMC11622614; doi:10.1080/21505594.2024.2435374)

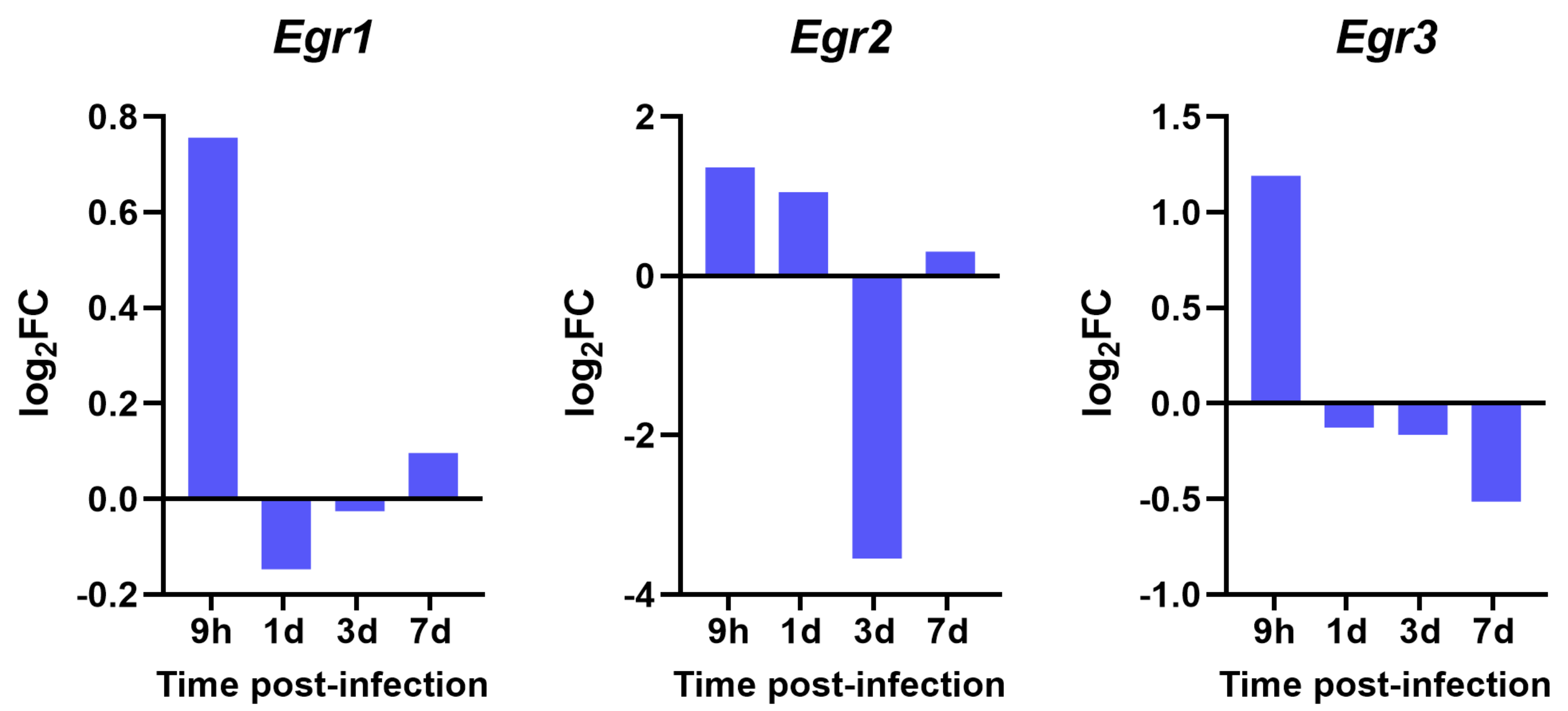

Supplement: Figure S1.tiff [file KVIR_A_2435374_SM2556.tiff]
